# Mean effective sensitivity for *Mycobacterium avium* subsp. *paratuberculosis* infection in cattle herds

**DOI:** 10.1186/s12917-015-0512-8

**Published:** 2015-08-08

**Authors:** Carsten Kirkeby, Kaare Græsbøll, Tariq Halasa, Nils Toft, Søren Saxmose Nielsen

**Affiliations:** National Veterinary Institute, Technical University of Denmark, Bülowsvej 27, DK-1870 Frederiksberg C, Denmark; Department of Applied Mathematics and Computer Science, Technical University of Denmark, DK-2800 Lyngby, Denmark; Department of Large Animal Sciences, University of Copenhagen, Grønnegårdsvej 8, DK-1870, Frederiksberg C, Denmark

**Keywords:** Paratuberculosis, Age-specific sensitivity, Control programme, Effective sensitivity, Milk ELISA

## Abstract

**Background:**

*Mycobacterium avium* subsp. *paratuberculosis* (MAP) infections in cattle are generally challenging to detect and cost-effective test strategies are consequently difficult to identify. MAP-specific antibody ELISAs for milk and serum are relatively inexpensive, but their utility is influenced by a number of factors such as herd size, herd composition and diagnostic sensitivity. The sensitivity of the test increases with the age of the tested animal, and therefore the general, or “mean effective sensitivity” (defined as the mean of the sensitivities for all animals within a population, MES), for detecting MAP within a herd is dependent upon the age distribution of the herd. For this study we used a dataset of cattle from 4,259 dairy herds and 4,078 non-dairy herds. The aim was to investigate the MES for groups of cattle considered to be reasonable entities for MAP surveillance and control, in order to assist the decision-makers in planning and optimizing these programs economically. We compared six different groups of cattle (three dairy and three non-dairy) in Denmark by calculating the MES for each herd in each group.

**Results:**

The distribution of MES showed a large variation within and between groups, and in some groups we found a bimodal distribution of MES. Dairy herds generally showed higher MES than non-dairy herds. Dairy herds in a control programme for paratuberculosis showed a MES similar to all other dairy herds from which animals > 2.0 years were tested (both groups had a median MES = 0.60). For the non-dairy groups, the sensitivity became much higher when animals < 2.0 years and herds with less than 25 cattle were excluded, resulting in a median MES of 0.65.

**Conclusion:**

The results showed that MES could indicate the effectivity of testing different cattle groups for MAP, given that the data used are unbiased.

## Background

*Mycobacterium avium* subsp. *paratuberculosis* (MAP) is a cause of financial loss to cattle farmers [1], and control is severely hampered by a long and variable incubation period, affecting the timely detection of infected or infectious animals. The most common diagnostic tests are associated with high costs or low performance, and their sole use in controlling MAP in cattle herds as part of a test-and-cull strategy is not generally advised [2–4]. Nonetheless, use of diagnostic testing, either alone or in combination with other means of disease management, does appear to enable a significant reduction in within-herd prevalences [5].

The diagnostic test sensitivity of ELISA for detection of infectious animals has been estimated at approximately 30 % [6], although it is affected by the test brand, the chosen cut-off value, and corresponding specificity among other factors, and the sensitivity increases if the animals are tested soon after they begin to shed [7]. However, if we set the target condition as infected adult cattle, the diagnostic sensitivity usually ranges between 5 % and 30 % for ELISA and culture [7], with significant variation between tests and studies. The herd-specific diagnostic sensitivity describes the probability of detecting MAP within a specific herd. This will depend on the age distribution within the herd, which may vary between different herds and production types such as dairy and non-dairy cattle [5, 8, 9]. Furthermore, the “true” and unbiased diagnostic sensitivity estimates often reported (summarised in [7]) for MAP-infected animals may be lower than the “effective” sensitivity, if the life expectancy of the individual is shorter than the time where the sensitivity is maximized in the population. We here introduce the term mean “effective” sensitivity (MES) as a measure of the sensitivity within a herd. MES is a function of the age distribution in the herd, and can be based on previously estimated age-specific sensitivities [9].

Surveillance programs for MAP can be focused on estimation of the prevalence on regional or national level, or on testing freedom of disease in an area. These estimates depend on the diagnostic sensitivity, which may depend on the sampling strategy, in particular if there is huge variation in the age-distribution. Design of sampling strategies is thus dependent on the expected test sensitivity in the study population, which can be measured with MES. Furthermore, targeted surveillance of specific subpopulations can be a cost-effective element in surveillance programs, while optimizing the coverage simultaneously with reducing the costs. MES can thus be a useful tool to describe the strength of studying a subpopulation, using for instance simulation modelling, so that the subpopulation with the highest MES can be chosen for surveillance. The objective of this paper was to estimate the distribution of MES in six different groups of cattle in Denmark. We stratified the cattle population into groups by the herd size and type (dairy and non-dairy), the age of each animal and by enrolment in the current MAP control programme. We also wanted to investigate the within-group as well as the between-group variation in MES and discuss the challenges in estimating the MES for these groups from available databases.

## Results

**Fig. 1:**
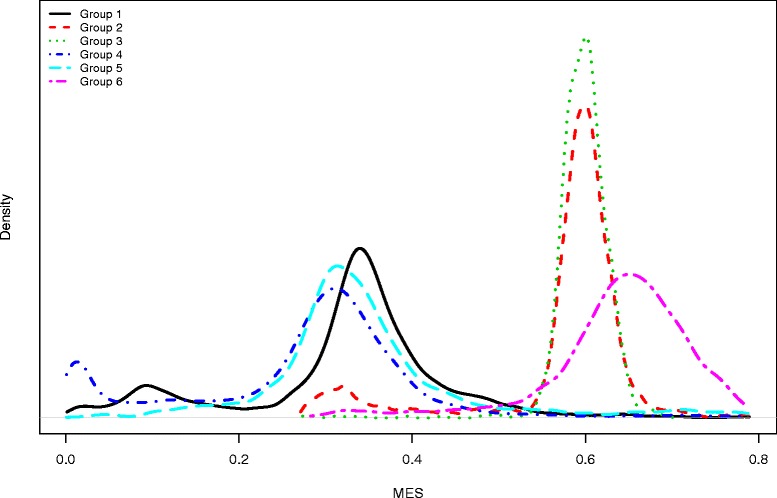
Density distributions of mean effective sensitivity (MES) calculated for different cattle subpopulations. Dairy herds: Group 1 = all cattle; Group 2 = all cattle > 2 years; Group 3 = all cattle above 2 years of age in the control programme. Non-dairy herds: Group 4 = all cattle in herds with at least 25 animals; Group 5 = all cattle in herds with at least 25 animals that were above 2 years of age; Group 6 = all cattle at least 2 years of age in herds with more than 25 animals that were at least 2 years of age

A total of 1,586,725 cattle were present in Denmark on the sampling date, 1,165,004 of which were from 4,295 herds recorded as dairy herds, 344,259 cattle were from 4,078 herds recorded as non-dairy herds with more than 25 animals, and the remaining 77,462 cattle were from non-dairy herds not assessed in this study. The age distributions for the six populations are shown in Table 1, while the resulting distributions of the MES in the herds are shown in Table 2 and Fig. 1. The median MES for each of the six groups were 0.34, 0.60, 0.60, 0.30, 0.33, and 0.65 respectively. The group with the lowest median MES was Group 4 - all cattle in non-dairy herds with more than 25 animals. The median MES of this group was only slightly lower than that of the groups with all cattle in Danish dairy herds and all cattle in non-dairy herds with more than 25 animals > 2.0 years of age. The group with the highest median MES was the group with all cattle > 2.0 years in dairy herds with more than 25 animals > 2.0 years. Generally, the groups with animals older than 2.0 years (Groups 2, 3 and 6) had a higher median MES than the groups where young livestock are included.

**Table 1:** Age distribution in the six cattle groups on 28 July 2014

Population	Number of		Age distribution (years)							
	Herds	Animals	Min	P5	Q1	Median	Q3	P95	P99	Max
1	4259	1,165,004	0.0	0.1	0.9	2.1	3.7	6.1	8.0	20.4
2	4082	605,945	2.0	2.1	2.7	3.6	4.8	6.9	8.7	20.4
3	949	188,832	2.0	2.1	2.7	3.6	4.8	6.9	8.7	19.0
4	4078	344,259	0.0	0.2	0.4	0.9	2.3	8.2	12.3	29.3
5	1345	139,851	0.0	0.2	0.6	1.5	4.2	9.4	13.4	27.1
6	1342	61,662	2.0	2.2	3.0	4.5	7.2	11.4	15.2	27.1

Dairy herds: Group 1 = all cattle; Group 2 = all cattle > 2 years; Group 3 = all cattle above 2 years of age in the control programme

Non-dairy herds: Group 4 = all cattle in herds with at least 25 animals; Group 5 = all cattle in herds with at least 25 animals that were above 2 years of age; Group 6 = all cattle at least 2 years of age in herds with more than 25 animals that were at least 2 years of age

**Table 2:** Distributions of the mean effective sensitivity (MES) of the ID Screen® *Mycobacterium avium* subsp. *paratuberculosis*-specific antibody ELISA

Population	Mean	Min	P5	Q1	Median	Q3	P95	Max
1	0.32	0.001	0.08	0.30	0.34	0.38	0.49	0.79
2	0.57	0.27	0.32	0.57	0.60	0.61	0.65	0.79
3	0.60	0.27	0.56	0.58	0.60	0.61	0.64	0.71
4	0.27	0.001	0.01	0.23	0.30	0.34	0.45	0.79
5	0.35	0.001	0.19	0.29	0.33	0.38	0.60	0.79
6	0.64	0.28	0.46	0.61	0.65	0.69	0.75	0.79

Dairy herds: Group 1 = all cattle; Group 2 = all cattle > 2 years; Group 3 = all cattle above 2 years of age in the control programme

Non-dairy herds: Group 4 = all cattle in herds with at least 25 animals; Group 5 = all cattle in herds with at least 25 animals that were above 2 years of age; Group 6 = all cattle at least 2 years of age in herds with more than 25 animals that were at least 2 years of age

## Discussion

This study is the first to provide distributions of mean herd sensitivity estimates for MAP infection in different populations. The MES calculated for a herd can be interpreted as the average sensitivity for a herd with a corresponding age-profile. The results are useful for optimizing MAP surveillance programs with regards to sensitivity, cost and number of samples to take from different groups. For example, a herd with a low MES may indicate that more samples should be collected from that herd in order to have the same information as would be obtained using fewer samples in a herd with a high MES. MES is also a useful way to measure the strength of a surveillance strategy. For example, we found that a relatively high MES (median 0.60) was obtained when testing animals above 2.0 years of age in dairy herds but a higher MES (median 0.65) was obtained when testing animals above 2.0 years in non-dairy herds with more than 25 animals. This means that large non-dairy herds are easier to detect MAP infection in, and the sample size can consequently be reduced slightly compared to dairy herds. However, in general we can adapt the sample size to achieve a comparable herd-level sensitivity even if the age-distribution differs. MES is also useful to show the potential of testing subpopulations, which could be further tested with simulation models.

In all groups except Groups 2 and 3, the variation in the sensitivity distribution was fairly large. In Groups 1, 2 and 4 we found a bimodal distribution. We ascribe this (at least for the two first groups) to a bias in the database registration, resulting in some heifer-rearing herds being included in Group 2, adding to the within-group variation. Therefore, we suggest that MES should be used with caution, as it may not reflect the sensitivity of the intended group. This should also be considered when selecting herds for estimation of within-herd prevalences of MAP or other studies which rely on the sensitivity of the test [10]. When deciding whether or not to test a given herd, MES could be used as an indicator of the test sensitivity within the herd. However, a better approach would be to estimate the sensitivity distribution directly from the age distribution of a specific herd of interest, in order to estimate the effective sensitivity of the herd.

We found that the MES was slightly higher for dairy herds than for non-dairy herds (Group 1 vs Groups 4 and 5), partly due to a bimodal distribution of the MES for non-dairy herds caused by a large proportion of young cattle intended for beef production purposes. This proportion of young cattle may differ seasonally, and consequently the sensitivity distribution also differs seasonally. This is not the case for dairy herds, where in Denmark calving occurs throughout the year. When comparing only animals > 2.0 years from large beef cattle herds and dairy herds (Groups 2 and 6), the MES in the beef herds was generally higher, but with more variation (Fig. 1). This is caused by a large number of old breeding animals in the beef cattle herds. Therefore, a practical subset for testing beef cattle herds could be Group 6.

In practice, Groups 1, 4 and 5 can be deemed similar, with medians of the MES in the range 0.30-0.34; Groups 2 and 3 were relatively similar, with median MES at 0.60; while Group 6 had the highest median MES of 0.65 (Fig. 1 and Table 2). This is interesting because it shows that it is possible to choose subpopulations of non-dairy herds where the sensitivity is considerably high. Whether this higher mean is of practical interest, e.g. for using test-and-cull strategies, can be assessed through modelling the impact of these differences (e.g. in a herd simulation model), which is beyond the scope of this paper. However, the use of the “effective” herd sensitivities instead of constructed “true” sensitivities may alter the optimal control strategies. The “true” sensitivity is often estimated from a population with a higher age-distribution than the actual age-distribution of the population where the test is used [3, 4]. If the majority of animals in the population where the “true” sensitivity was estimated had a lifespan of greater than 5–6 years, where the sensitivity is maximized, then we underestimate the sensitivity. To avoid this "healthy worker survivor effect", it is necessary to include an age-distribution reflecting that in the target-population when estimating the sensitivity. Essentially, many animals are culled much earlier, and thus we avoid this underestimation in the use of “effective sensitivity”. A key assumption for the validity of the estimate is that the age distribution in the assessed population is similar to the age distribution of the infected cattle in the same population.

The only previous age-specific sensitivity estimates available are those presented in [9], which are used in the present study. The approach employed in that paper may impact on the accuracy of those estimates, which would have an impact on the results presented here. However, we argue that the MES could be a useful proxy since we are ultimately interested in using the average sensitivity in relation to how long an animal actually lives, and not how long it can potentially live. Therefore, the estimates of the sensitivities shown in this analysis are likely to be more realistic than the estimates from numerous test-evaluations summarised in [7]. The age distributions and factors impacting the sensitivity may, however, differ between countries. This means that although the principles are directly transferable to such populations, the results may not be. It should be noted that the sensitivity in [9] may have been overestimated because persistent latent infections were automatically excluded from that study.

When evaluating control programs for MAP in groups of cattle, it is also necessary to consider the test specificity [11]. The test specificity of ELISA, faecal culture and PCR are not entirely accurate for two primary reasons: a) false-positive reactions occur in immunological tests due to cross-reacting antibodies [12]; b) MAP may be excreted by non-infected animals in high-prevalence herds due to passive (pass-through) shedding of the bacteria [13, 14], or because infected but non-infectious cattle are detected by agent-detecting tests. Although beyond the scope of this study, it would be interesting to evaluate the test specificity in the same way as we have evaluated the impact of test sensitivity on actual data distributions. Even though an ideal test for a test-and-cull strategy would identify infected animals before they become infectious, it can be undesirable if early-stage infections are not the target of the testing (e.g. if only a subset of the infected animals will become infectious or diseased). The latter point is again related to the age distribution in a given herd, and could be included in a model simulating different tests. Therefore, we emphasize the importance of using herd-specific data in simulation modelling and decision-making.

## Conclusions

We coined the term “Mean Effective Sensitivity” (MES) to describe the herd-level sensitivity when testing for MAP. We calculated the MES for different subpopulations of cattle herds in Denmark and found a large variation between the investigated groups. Some groups had a bimodal distribution, but groups including young stock had low MES, whereas groups excluding young stock had on average high MES. However, the variation within a group was so large that it might be worthwhile to estimate the MES in a specific herd before embarking on a test scheme. The study confirms the general opinion that testing young stock is not beneficial. This updated information can be used to improve modelling of the effective sensitivity in mathematical and simulation models in order to optimize surveillance and control programs.

## Materials and methods

### Herds and animals

The animals recorded in the Danish Cattle Database on 28 July 2014 were used to assess the within-herd age distribution in the six populations in question. By law, the birth date of all cattle born in Denmark must be recorded along with the date of death, and these recordings were used to establish the number and age of live animals. The database also recorded whether or not the herd was producing milk, and we were therefore able to divide herds into dairy and non-dairy groups. As a consequence, the latter group consisted of herds rearing calves or young stock smallholders (“hobby” farmers), as well as regular beef herds. We defined six groups of interest to explore in this study: 1) all cattle in Danish dairy herds; 2) all cattle > 2.0 years in Danish dairy herds; 3) all cattle > 2.0 years in dairy herds enrolled in the Danish control programme on paratuberculosis [15]; 4) all cattle in non-dairy herds with more than 25 animals; 5) all cattle in non-dairy herds with more than 25 animals > 2.0 years of age; and 6) all cattle above 2.0 years of age in non-dairy herds with more than 25 animals > 2.0 years of age. The herd size and age cut-offs were selected arbitrarily. An age of 2.0 years was chosen as the definition of adulthood.

Testing young stock (< 2.0 years) by ELISA is generally discouraged due to low sensitivity (few animals will test positive as infection is still latent). Their inclusion in some of the groups in the present study was mainly motivated by a desire to determine the impact on the mean herd sensitivity compared to testing of adult cattle alone. We used all dairy cattle herds irrespective of herd size, since in Denmark they generally consist of > 25 animals, with the mean herd size in Group 2 being 149 adult cattle > 2.0 years in each herd.

### Age-specific sensitivity

Data for the MAP-specific ID Screen® (IDvet, Grabels, France) antibody ELISA used for milk samples was used, as relevant sensitivity and specificity estimates were available for this test. The effective test specificity has been estimated to 0.9866 [9], but this information was not specifically included. It is acknowledged that young stock and beef herds cannot be tested using milk samples, however it was assumed that the serum ELISA used on these animals would perform equally well [15–17], and that the estimates were valid for young stock as estimated. Since there is no reference standard test for MAP infection, we used the case definition of Nielsen et al. [9], defined as a cow being ELISA-positive following repeated testing with a minimum of two samples. However, it is important to note that we did not use test-results from actually tested cows in this study, but only used the existing age-distribution within the investigated groups.

The function for age-specific effective sensitivity used here has been described by Nielsen et al. (2013) and is defined as:

1 $$ Se\;\left( ag{e}_i\right) = \frac{e^{1.32 - 9.38\kern0.24em {e}^{-0.70\kern0.24em  ag{e}_i}}}{\left(1+{e}^{1.32-9.38\;{e}^{-0.70\kern0.24em  ag{e}_i}}\right)} $$

where *Se(age*_*i*_*)* is the sensitivity depending on the age of animal *i*. The herd mean effective sensitivity, MES, was then estimated by calculating the sensitivity for each cow in the herd using the function described above, and then taking the mean of the estimated sensitivities for all animals belonging to each group in each herd:

2 $$ ME{S}_{jk}=\frac{{\displaystyle {\sum}_i^{N_{jk}}Se\;\left( ag{e}_{ijk}\right)}}{N_{jk}} $$

So *MES*_*jk*_ is the mean effective sensitivity for group *j* within herd *k*, where *N*_*jk*_ is the total number of cows in group *j* within herd *k*, and *Se(age*_*ijk*_*)* is the sensitivity for animal *i* belonging to group *j* within herd *k*. Note that one herd may have cows belonging to multiple groups, and may therefore contribute several times to the results.

## Ethics statement

This study is theoretical and did not need to comply with any guidelines.

## Abbreviations

MAP: *Mycobacterium avium* ssp. *paratuberculosis*
MES: Mean effective sensitivity
